# Deep learning system for the auxiliary diagnosis of thyroid eye disease: evaluation of ocular inflammation, eyelid retraction, and eye movement disorder

**DOI:** 10.3389/fcell.2025.1609231

**Published:** 2025-06-16

**Authors:** Yu Han, Jun Xie, Xiaoyu Li, Xinying Xu, Bin Sun, Han Liu, Chunfang Yan

**Affiliations:** ^1^ College of Electronic Information Engineering, Taiyuan University of Technology, Jinzhong, Shanxi, China; ^2^ College of Electrical and Power Engineering, Taiyuan University of Technology, Taiyuan, Shanxi, China; ^3^ Department of Orbital Diseases, Shanxi Eye Hospital Affiliated to Shanxi Medical University, Taiyuan, Shanxi, China

**Keywords:** thyroid eye disease (TED), multi-label image classification, semantic segmentation, feature extraction, automatic quantization, eye digital image dataset

## Abstract

**Objective:**

This study aims to construct a semantic segmentation-based auxiliary diagnostic model for thyroid eye disease (TED) focusing on eyelid retraction, eye movement disorders, ocular inflammation related to Clinical Activity Score (CAS), facilitating rapid and non-invasive diagnosis for suspected TED patients and enhancing the efficiency of treatment and diagnosis.

**Methods:**

Data were collected from 153 subjects exhibiting symptoms of eyelid retraction, eye movement disorders, and ocular inflammation related to CAS. After quality screening, datasets for the primary position (303 eyes), gaze positions (1,199 eyes), and a multi-label inflammatory classification dataset (272 eyes) were constructed. The constructed TBRM-Net adopts a dual-branch feature extraction and fusion strategy to extract inflammation features for multi-label classification and recognition; the constructed DSR-Net performs segmentation of ocular structures and has designed a quantitative diagnostic algorithm.

**Results:**

The semantic segmentation-based auxiliary diagnostic model for TED demonstrated a mean pixel accuracy (MPA) of 94.1% in the primary position dataset and 95.0% in the gaze positions dataset. The accuracy for diagnosing eye movement disorders, upper eyelid retraction, and lower eyelid retraction reached 85.4%, 95.1%, and 87.0%, respectively. The accuracy for Redness of Eyelids, Swelling of Eyelids, Redness of Conjunctiva, Swelling of Conjunctiva, and Swelling of Caruncle or Plica reaches 81.8%, 78.8%, 90.6%, 73.5%, and 83.9%, respectively, with an average accuracy of 81.7%. Segmenting and classifying images of structures affected by ocular inflammation can effectively exclude interfering features. The designed quantitative algorithm provides greater interpretability than existing studies, thereby validating the effectiveness of the diagnostic system.

**Conclusion:**

The deep learning-based auxiliary diagnostic model for TED established in this study exhibits high accuracy and interpretability in the diagnosis of ocular inflammation related to CAS, eyelid retraction, and eye movement disorders. It holds significant medical value in assisting doctors in formulating treatment plans and evaluating therapeutic effects.

## 1 Introduction

Thyroid eye disease (TED), also known as Graves’ ophthalmopathy (GO), is a type of autoimmune inflammatory orbital disease that ranks first in the incidence of adult orbital diseases (Oculoplastic and Orbital Disease Group of Chinese Ophthalmological Society of Chinese Medical Association et al., 2022; Barrio-Barrio et al., 2015; Peter, 2012). The male to female prevalence ratio of TED is approximately 2:3, with a higher incidence in females across the general population; however, in the elderly, the prevalence is higher in males than in females (Zhang, 2023). The clinical manifestations of TED are complex and variable, primarily affecting the eyelids, extraocular muscles, and orbital adipose tissue, leading to eyelid abnormalities, eye movement disorder, and even compressive optic neuropathy (Ou et al., 2022). Eyelid abnormalities in TED primarily manifest as retraction of the upper and lower eyelids, which is one of the most common signs of the condition and may even lead to exposure keratopathy. Inflammatory signs include redness of the eyelids, swelling of the eyelids, redness of the conjunctiva, conjunctival edema, and inflammation of the caruncle and/or plica. TED involves four extraocular muscles, with males experiencing more frequent involvement than females, and the severity increasing with age (Zhang, 2023), resulting in disturbances of eye movement. The traditional method for assessing eyelid position in TED involves clinicians using a ruler for measurements and an examination light to observe the patient’s eye movements.

There are established diagnostic criteria and management guidelines for this disease (Oculoplastic and Orbital DiseaseGroup of Chinese Ophthalmological Society of Chinese MedicalAssociation et al., 2022). Eyelid retraction is one of the primary signs for diagnosing TED. The diagnosis of eyelid retraction and eye movement disorder requires the collaboration of experienced clinicians and highly cooperative patients. This collaboration is essential for accurately diagnosing and devising treatment plans to mitigate or even prevent disease progression. However, there is a scarcity of specialists in orbital diseases within the country, and doctors in remote areas often lack experience in diagnosing and treating TED. Consequently, TED is prone to misdiagnosis and mistreatment, particularly in the early stages of the disease. Developing a deep learning-based method for automatic segmentation of ocular morphology in suspected TED cases with concurrent quantitative assessment of eyelid retraction and eye movement disorders, along with automated detection of Clinical Activity Score (CAS)-associated inflammatory signs (Redness of Conjunctiva, Redness of Eyelids, Swelling of Coniunctiva, Swelling of Eyelids, Swelling of Caruncle or Plica), can assist clinicians in TED diagnosis, standardized staging, and therapeutic optimization. This approach assists clinicians in diagnosing TED, enhancing diagnostic efficiency. By combining assessments of eyelid retraction and ocular motility impairment with the CAS, this method provides accurate grading of TED severity (Oculoplastic and Orbital Disease Group of Chinese Ophthalmological Society of Chinese Medical Association et al., 2022). This approach holds significant value in formulating treatment plans and evaluating therapeutic efficacy for patients.

In recent years, the advantages of deep learning technology in image processing have been instrumental in aiding the diagnostic imaging of TED (Diao et al., 2023; Shao Y. et al., 2023). Xiao et al. (2022) conducted a classification study based on facial images to identify eye movement disorders and eyelid retraction, but the interpretability was limited. Justin et al. (2022) developed an artificial intelligence platform based on a deep learning (DL) model that recognizes the presence of TED through ocular photographs and generates heatmaps to represent pathological areas within facial images. Jae Hoon et al. (2022) used facial images to assess the CAS and predict disease activity. Shao Ji et al. (2023) utilized a deep learning-based analysis system to automatically calculate comprehensive morphological parameters of the eyelids, such as palpebral fissure (PF) length and eyelid retraction distance, to quantify ocular parameters without further diagnostic interpretation. In terms of ocular structure segmentation, Chen et al. (2023) employed a lightweight algorithm to segment the sclera, eyelids, and lacrimal caruncle areas in TED patients. Naqvi et al. (2020) used SegNet as the backbone network for the segmentation study of the sclera and iris. Additionally, Hu et al. (2022) diagnosed eyelid retraction based on regional Hough transform, and Liang (2021) built upon this for the grading diagnostic study of TED.

This study designed a deep learning system for multi-label inflammatory classification of TED symptoms and quantitative assessment of eyelid retraction and eye movement disorders, enabling rapid, non-invasive preliminary screening. The main work includes three aspects:

(1) Facial photographs of study subjects were collected, and under physician guidance, we constructed both primary position and gaze positions datasets (153 subjects including 100 TED patients) for quantitative diagnosis of eyelid retraction and eye movement disorders. A multi-label structured dataset was built to classify five CAS-related symptoms.
(2) For the multi-label structured dataset, we performed targeted cropping of the eyelid structure, conjunctival structure, and lacrimal mound structure to eliminate irrelevant inflammatory feature interference. This preprocessed data was integrated into our classification algorithm, and we have developed a dual-branch feature fusion network, TBRM-Net, which is based on MobileViT and residual blocks, for ocular inflammation feature extraction to achieve higher classification accuracy.
(3) To address quantification challenges in the primary position and gaze positions datasets, we developed a DSR-Net model for semantic segmentation of ocular structures. By incorporating clinical standard-based quantification algorithms with reference scales, we achieved quantitative evaluation of both eyelid retraction (including severity degree) and eye movement disorders (including impairment extent).

## 2 Materials and methods

### 2.1 Data collection

Data in this article were collected from 153 patients with thyroid ophthalmopathy (TED) and normal controls, including 100 TED patients (51 females) with an average age of 49.5 ± 26 years, and 53 normal controls (33 females). This study was approved by the Ethics Review Committee of Shanxi Eye Hospital Affiliated to Shanxi Medical University and was conducted in accordance with the principles of the Declaration of Helsinki. Due to the retrospective design of the study, only prior medical records and facial photographs of patients were used, and all facial photographs were anonymized and blurred to obscure identifiable features. Consequently, the Ethics Review Committee waived the requirement for informed consent. All experiments were performed in accordance with relevant guidelines and regulations. Personal information was removed from all images and clinical data before external processing to ensure strict confidentiality. The data transmission process was approved by the Ethics Review Committee.

Based on the standardization of data collection methods, all facial photographs of the study subjects were taken under natural lighting conditions using a dedicated smartphone (model Honor 20 Pro) mounted on a special stand at a distance of 25 cm, with a resolution of 2340 × 1080 pixels. The smartphone used was equipped with a 48-megapixel rear main camera (aperture f/1.4), capable of high-quality image acquisition and supporting various shooting modes. Considering the impact of head stability on image quality and subsequent research, a specialized head fixation device was designed to meet the photography requirements. This device, inspired by the slit-lamp stand, features a base and a head positioning frame, with a forehead support at the top and a chin rest at the bottom. The side vertical rod is an adjustable mechanism that can be tailored to the patient’s facial shape. A visual target is set above the head fixation frame to assist in capturing five-position facial photographs. The side of the device is equipped with a scale (alternating black and white, each division representing 5 mm), which serves as a reference for image size restoration during data processing, providing a basis for quantitative recognition. The use of a dedicated photography device ensures consistency and stability of the images during shooting.

The contours for localizing the eye region and drawing the scale bar were manually delineated using the open-source interactive software tool LabelMe. The cropped eye images from the primary position, obtained through Model 1, were annotated for the sclera, iris, pupil, and lacrimal caruncle and plica regions (LCP). Similarly, the cropped eye images from the gaze position, also obtained through Model 1, were annotated for the sclera, iris, and pupil. These annotations were used to quantify the diagnosis of TED-related Eyelid retraction and eye movement disorder. The annotated areas were then mapped using one-hot encoding in the annotation maps.

The processed multi-label inflammatory classification dataset includes 278 eyes comprising eyelid structure, conjunctival structure, and LCP structure. While the semantic segmentation datasets included 272 eyes in primary position and 1,075 eyes in gazes position. Notably, the primary position dataset contained 99 eyes with upper eyelid retraction and 115 with lower eyelid retraction. Both healthy volunteers and TED patients were randomly allocated into training, validation, and test sets at an 8:1:1 ratio for model development. The data distribution of each symptom and the sample data after data enhancement is shown in Table 1.

**TABLE 1:** Data distribution.

Statistical Data	Eyelid Inflammation		Conjunctival Inflammation		LCP	Primary position	Gaze positions
	Redness of eyelids	Swelling of eyelids	Redness of conjunctiva	Swelling of coniunctiva	Swelling of caruncle or plica	Eyelid retraction	Eye movement disorder
Quantity	74	186	182	50	102	115	341
Account for	26%	67%	65%	18%	37%	42%	32%
Enhancement	222	186	364	200	306	1,360	1705

### 2.2 Diagnosis method

**FIGURE 1:**
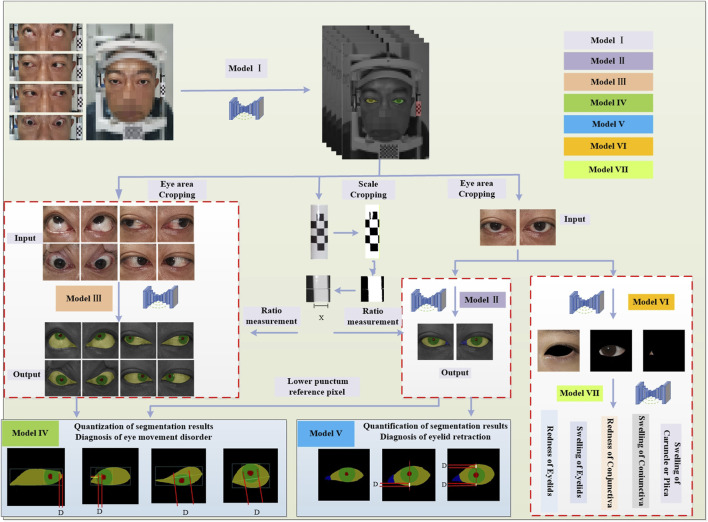
Diagnostic system framework. The system framework comprises Module I for detecting the eye region and the drawing scale bar, Module II for identifying ocular structures in the primary position, Module III for identifying ocular structures in gaze positions, Module IV for the quantitative diagnosis of eye movement disorder, Module V for the quantitative diagnosis of Eyelid retraction, Module VI for the multi-label classification structure cropping, and Module VII for the structural inflammation classification.

This study constructs an AI-assisted diagnostic system based on clinical diagnostic criteria. The system includes the following modules: the module for localizing the eye region and drawing the scale bar (Module I), the semantic segmentation module for the primary position (Module II), the semantic segmentation module for gaze positions (Module III), the module for quantitative diagnosis of eye movement disorder (Module IV), and the module for quantitative diagnosis of Eyelid retraction (Module V), the multi-label classification structure cropping module (Module VI), and the structural inflammation classification module (Module VII). The system offers high interpretability in its diagnostic process, as illustrated in Figure 1.

In Module I, the entire facial image of the patient is input into the trained DSR-Net, which analyzes the weights to localize the eye region and the drawing scale bar. The eye region is cropped to a size of 512 × 512, and the drawing scale bar is processed to obtain the actual length.

Module II and Module III perform semantic segmentation on the primary position dataset to identify four structures: sclera, iris, pupil, and lacrimal caruncle and plica (LCP), and on the gaze positions dataset to identify three structures: sclera, iris, and pupil, respectively. The structural boundaries of the ocular images obtained from these modules are then passed to Module IV and Module V for further analysis.

Module IV is a quantitative model for diagnosing eye movement disorder, which constructs distinct diagnostic logic based on the presence or absence of movement impairments in four directions: upward, downward, leftward, and rightward. In both Module IV and Module V, D represents the pixel reference length, and the quantitative results are derived by combining D with the ratio measurement obtained from the drawing scale bar.

Module V is a quantitative model for diagnosing Eyelid retraction, using the vertical line passing through the center of the pupil as a reference. The diagnostic logic is constructed based on four levels: the presence of only upper eyelid retraction (scleral exposure above the vertical line of the pupil); the presence of both upper and lower eyelid retraction (scleral exposure above and below the vertical line of the pupil); the presence of only lower eyelid retraction (scleral exposure below the vertical line of the pupil); the absence of eyelid retraction.

Module VI performs targeted cropping of the eyelid, conjunctival, and lacrimal-caruncle-plica (LCP) structures from primary position, with the cropped structural images corresponding to specific inflammatory symptom labels for subsequent multi-label classification in Module VII.

Module VII implements a multi-label classification network that adopts a dual-branch feature extraction and fusion strategy to comprehensively characterize inflammatory features, enabling precise multi-label classification of inflammation corresponding to specific ocular structures.

### 2.3 Quantitative standard

**FIGURE 2:**
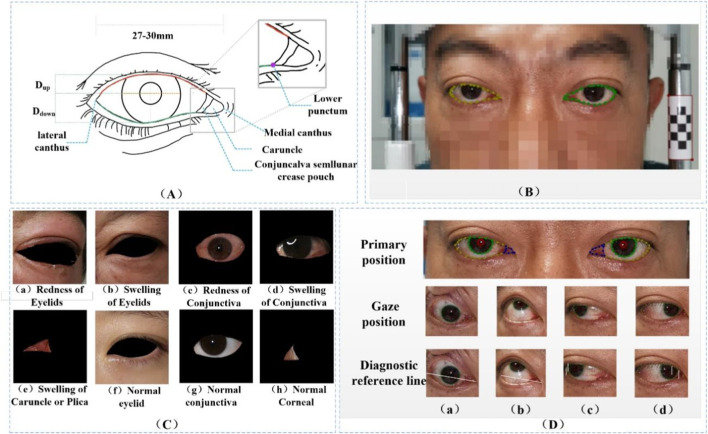
**(A)** Eye structure. D_up_ and D_down_ are the distances from the margin of the upper eyelid and the margin of the lower eyelid to the center of the pupil, respectively, to assist in the diagnosis of eyelid retraction. **(B)** The annotation of eye and drawing scale for facial image recognition. **(C)** Structural data annotation for multi-label classification. **(D)** Quantitative diagnosis of eyelid retraction and eye movement disorders involved in eye structure labeling.

The eye structure is shown in Figure 2A. D_up_ and D_down_ are the distances from the margin of the upper eyelid and the margin of the lower eyelid to the center of the pupil, respectively, to assist in the diagnosis of eyelid retraction. Figure 2B is the annotation of eye and drawing scale for facial image recognition. Figure 2C shows the structural data of multi-label classification. Figure 2D shows the semantic segmentation annotation of eye structure.

In current clinical diagnostic practice, the criteria for diagnosing eye movement disorders (Yang and Fan, 2018) and for assessing eyelids (Bartalena et al., 2016) are clear. This paper translates these criteria into quantifiable thresholds for the purpose of conducting research. The reference point of the medial canthus is converted to the medial point of the caruncle, and the reference point of the lateral canthus is transformed to the lateral point of the sclera. The reference position of the lower lacrimal punctum in the clinical standard for medial gaze positions (Tao, 2019) is altered to the intersection point of the lateral side of the fold region with the lower eyelid margin (Figure 2A), which facilitates the quantification of eyelid retraction in facial images and the quantitative diagnosis of eye movement disorders. Under the guidance of ophthalmologists at the Shanxi Eye Hospital, annotations of ocular structures are performed, with the caruncle and fold region combined for annotation to facilitate subsequent research. The conversion of diagnostic criteria is as follows:

(1) Upward or Downword: Coincidence of the iris with the line connecting the medial and lateral canthi, with an overlap of less than 20 pixels, is considered normal (approximately 1.4 mm, as confirmed by actual diagnostic results).
(2) Right eye left or Left eye right: The distance from the inner margin of the pupil to the medial canthus minus the distance from the lower punctum lacrimale to the medial canthus being less than 0 pixels is indicative of a normal condition.
(3) Left eye left or Right eye right: The distance from the outer margin of the iris to the lateral canthus being less than or equal to 0 is considered normal.
(4) Upper eyelid retraction: Taking the center of the pupil as the reference vertical line, when the corneal area is relatively well exposed, the distance from the lower palpebral margin to the center of the pupil minus the distance from the upper palpebral margin to the center of the pupil is greater than 1 mm, which is considered normal.
(5) The distance from the lower eyelid margin to the inferior margin of the cornea being less than 0 mm is considered normal.

**FIGURE 3:**
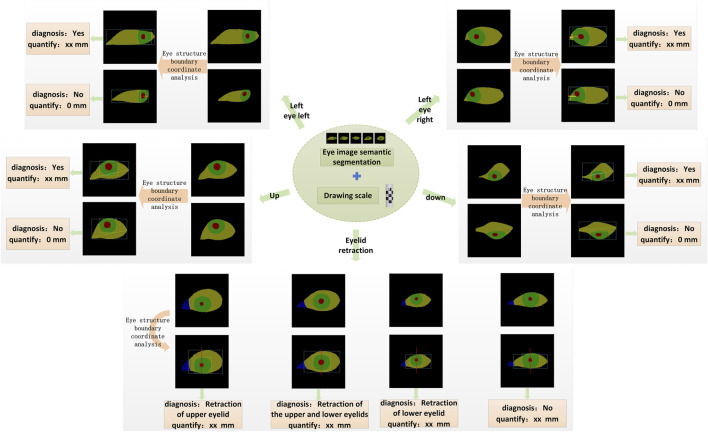
The schematic diagram of the quantitative diagnostic model is shown. Based on the quantitative results, each gaze direction can be classified into movement disorder or normal movement. Similarly, Eyelid retraction is categorized into four levels according to the diagnostic logic: upper eyelid retraction, both upper and lower eyelid retraction, lower eyelid retraction, and normal.

In this study, quantitative diagnosis of eyelid retraction and eye movement disorder is conducted based on the semantic segmentation results, with the aid of auxiliary lines and a scale bar. The boundary coordinates of the segmented ocular structures are analyzed and input into different diagnostic logics, as illustrated in Figure 3.

### 2.4 Model framework

The architectural framework of the semantic segmentation network model DSR-Net is responsible for the operational integration of Modules I, II, III, and VI. The DSR-Net primarily consists of five components: the feature encoder module, the feature decoder module, the SE-Block, the Dense Atrous Convolution (DAC) block, and the Residual Multi-kernel Pooling (RMP) module. Initially, the image is processed by the feature encoder module, which preliminarily extracts features and reduces the spatial dimensions of the feature map through a 7 × 7 convolution with a stride of 2, followed by max pooling downsampling and residual blocks for feature extraction and encoding. Subsequently, the DAC block and RMP module are employed to extract features of targets at various scales and global contextual information. The decoder then progressively restores the feature maps from the encoder (processed by the SE module) and the high-level semantic feature maps extracted by the DAC and RMP modules to the original size of the segmentation result image through four upsampling steps using deconvolution.

**FIGURE 4:**
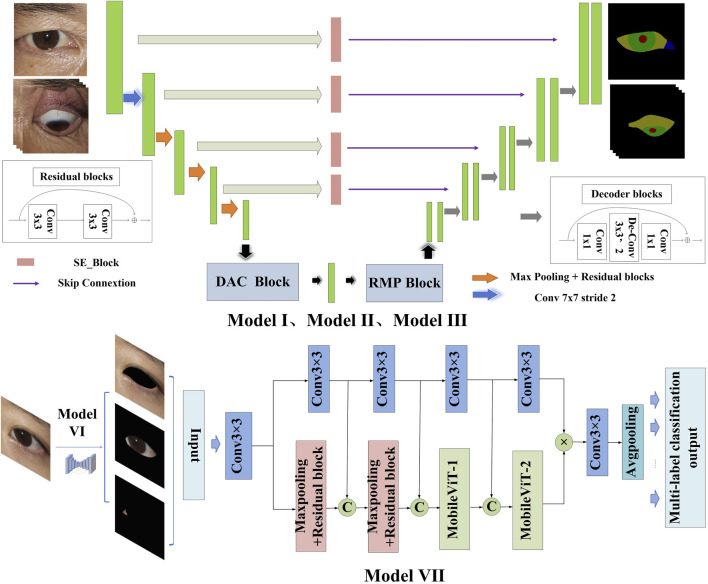
Model structure.

The multi-label classification network TBRM-Net, responsible for Module VII, employs a dual-branch feature fusion strategy. The input images are inflammatory structure segmentation maps. For eyelid structure images, the network classifies labels into Redness of Eyelids, Swelling of Eyelids, and normal; for conjunctival structure images, it classifies labels into Redness of Conjunctiva, Swelling of Coniunctiva, and normal; and for LCP structures, it classifies labels into Swelling of Caruncle or Plica and normal. The first branch of the input image utilizes the MobileViT module to initially extract features through max pooling and residual connections, and then delivers the extracted features to the MobileViT module for further extraction of global features. The second branch employs 3 × 3 convolutional blocks to extract high-dimensional features from the image, and at each layer, these features are concatenated and fused with those extracted from the first branch. The features from both branches are multiplied to achieve feature fusion. Finally, the fused features undergo channel dimension reduction through a 3 × 3 convolution, followed by global average pooling to compress the feature map, and are then flattened to output the results of the multi-label classification head. The model structure is shown in Figure 4.

The DAC block incorporates four cascaded branches with dilated convolutions of dilation rates 1, 3, and 5, respectively. In each dilated convolution branch, a 1 × 1 convolution followed by ReLU is applied. The branch with a larger receptive field extracts features of large-sized targets, while the branch with a smaller receptive field extracts features of small-sized targets. By combining dilated convolutions with different dilation rates, the DAC block captures features of targets across various scales, thereby acquiring more comprehensive and deeper semantic information.

The SE module (Squeeze-and-Excitation module) utilizes global average pooling to compress the input feature maps. By learning the inter-channel weight relationships through different compression ratios r, it generates a channel-wise weight vector with values ranging between [0, 1]. These weights are then multiplied by each channel of the original input features X, thereby recalibrating the importance of each channel. The channel weight vector s is expressed as shown in Equation 1.

$$s=F\left(z,W\right)=\sigma \left(g\left(z,W\right)\right)=\sigma \left(FC_{2}\delta \left(FC_{1}Z\right)\right)$$ (1)

$$\tilde{X}=s\cdot X$$ (2)

FC_1_ represents a fully connected layer that reduces the output to C/r channels; δ denotes the ReLU activation function; FC_2_ is a fully connected layer with an output of C channels; σ signifies the Sigmoid function, $\tilde{X}$ is the output feature map, as shown in Equation 2.

**FIGURE 5:**
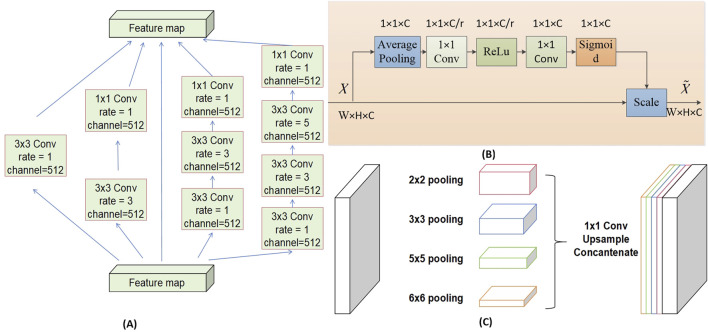
**(A)** DAC _Block structure, **(B)** SE _Block structure and **(C)** RMP_Block structure of DSR-Net.

The RMP block encodes multi-scale contextual features of objects extracted from the DAC module by employing pooling operations of various sizes. It utilizes four branches with different receptive fields to encode global contextual information, each branch outputting features of different scales. After each scale-specific feature, a 1 × 1 convolution is applied to reduce the feature map size to 1/N of the original size. The low-dimensional feature maps are then upsampled to match the size of the feature maps output by the DAC module. Finally, the multi-scale features are concatenated with the features extracted by the DAC module along the channel dimension for subsequent decoding. The structures of each Block are illustrated in Figure 5.

### 2.5 Implementation

The experiment was conducted on the integrated development environment PyCharm 2022.3.2 (JetBrains Inc., Czech Republic), using the computer programming language Python 3.8 (Guido van Rossum, Netherlands), and the deep learning framework PyTorch 1.12.1 - cu113 (Facebook Inc., United States). The experiment utilized the processor Intel^®^ Xeon^®^ Silver 4210R CPU @ 2.40 GHz (Intel Inc., United States) and the graphics card NVIDIA GeForce RTX 3090 Ti GPU (Nvidia Inc., United States).

This paper involves the localization and annotation of facial images (eye position and drawing scale) and the annotation of ocular structures. The training model achieves eye positioning and simultaneously crops the labeled images to a size of 512 × 512 pixels, ensuring the complete display of ocular structures and facilitating network input processing. Data augmentation is performed on the eye appearance maps, which includes horizontal flipping, brightness adjustment with a parameter of 1.5, contrast adjustment with an alpha of 1.5 and a beta of 0, dilation and erosion using 3 × 3 convolution kernels, and a probabilistic deflection within the range of (−20°, +20°) at a probability of 0.5. This approach simulates the illumination variations and individual differences in eye growth observed during actual examinations. The network employs the adaptive moment estimation (Adam) optimizer, with an initial learning rate of 5 × 10^−4^, which is gradually decreased in subsequent epochs. The parameter r in the SE module is set to 16. The batch size is set to 8, and the training is conducted for 200 epochs.

The network is optimized using a cross-entropy loss function. By minimizing the discrepancy between the predicted probabilities and the actual labels, the model is encouraged to learn more accurate segmentation; the higher the precision of the prediction results, the lower the loss value. In this experiment, different weights are assigned to various eye regions, aiming to give more learning emphasis to important structures. For the primary position, the loss function weights were set to (1, 4, 4, 8), corresponding to the sclera, iris, pupil, and lacrimal caruncle and plica (LCP) regions, respectively. For the gaze positions, the loss function weights were set to (1, 4, 8), corresponding to the sclera, iris, and pupil regions, respectively. Equation 3 represents the loss function formula.

$$L=-\sum_{i=1}^{N}\sum_{c=1}^{C}w_{c}y_{i,c}\log\left(p_{i,c}\right)$$ (3)

N represents the batch size, C is the number of classes, yi,c is one if sample i belongs to class C, and 0 otherwise, pi,c is the probability that the model predicts sample i belongs to class C. Wc is the weight for each class C, set to different parameters to enhance learning for diagnostically important areas, such as the pupil and other regions.

The model’s diagnostic performance was evaluated using the Python 3.8 programming language, with metrics such as IoU (Intersection over Union, Equation 4), Dice coefficient (Equation 5), and MPA (Mean Pixel Accuracy, Equation 6) to assess the segmentation effectiveness. Both are used to measure the similarity between the network’s segmentation results and the gold standard. The diagnostic analysis results were evaluated using ACC (accuracy, Equation 7), P (precision, Equation 8), and TPR (true positive rate, Equation 9). i and j represent the target and non-target classes (such as the pupil region and non-pupil region), or the positive and negative outcomes of the diagnostic results, respectively. p_ii_ denotes the correctly segmented pixels, which are true positives, while p_ij_ and p_ji_ represent false positives and false negatives, respectively. k is the number of segmentation classes.

$$IoU=\frac{p_{ii}}{p_{ij}+p_{ji}+p_{ii}}$$ (4)

$$Dice=\frac{{2p}_{ii}}{p_{ij}+p_{ji}+2p_{ii}}$$ (5)

$$MPA=\frac{1}{K+1}\sum_{i=0}^{k}\frac{p_{ii}}{p_{ij}+p_{ji}}$$ (6)

$$Acc=\frac{p_{ii}+p_{jj}}{p_{ij}+p_{ji}+p_{ii}+p_{jj}}$$ (7)

$$P=\frac{p_{ii}}{p_{ij}+p_{ii}}$$ (8)

$$TPR=\frac{p_{ii}}{p_{ji}+p_{ii}}$$ (9)

## 3 Results

This experiment introduces classic advanced deep learning models from the fields of natural and medical imaging, comparing eight models in semantic segmentation task: U-Net (Ronneberger et al., 2015), SE-UNet (Hu et al., 2018), SegNet (Badrinarayanan et al., 2017), DeepLab v3 (Chen et al., 2019), UNeXt (Valanarasu and Patel, 2022), ResNet (Xie et al., 2016), Deep Pyramid (Johnson and Zhang, 2016), and CE-Net (Gu et al., 2019). The DSR-Net was trained on the primary position dataset and the gaze positions dataset, and the weights with the highest Mean Pixel Accuracy (MPA) on the validation set were selected for testing on the test set. In the multi-label classification task, the structural datasets Eyelid structure dataset, Conjunctival structure dataset, and LCP dataset were used to compare the accuracy of the proposed model with classical models such as Resnet (Xie et al., 2016), Resnet50 (Xie et al., 2016), ConvNet (Liu et al., 2022), ShuffleNet_v2 (Ma et al., 2018), MobileNetv2 (Sandler et al., 2018), and MobileVit (Mehta and Rastegari, 2021). The weights with the highest accuracy on the validation set were selected for testing. The results demonstrate that the two models proposed in this paper exhibit high segmentation precision and classification accuracy, thereby facilitating clinical auxiliary diagnosis.

In the tasks of Model I and Models II and III, the facial localization results are presented in Table 2. The evaluation metrics IoU and MPA for the segmentation effectiveness of ocular structures in the tasks of primary position segmentation and gaze position segmentation are detailed in Table 3 respectively. The Dice coefficient is presented as shown in Table 4.

**TABLE 2:** Facial location results.

Structure	IoU	Dice
Left eye	0.811	0.894
Right eye	0.801	0.882
Drawing scale	0.881	0.934

**TABLE 3:** Segmentation results of each model (IoU/MPA).

Model	Primary position					Gaze positions			
	Scleral	Iris	Pupil	LCP	MPA	Scleral	Iris	Pupil	MPA
U-Net	0.855	0.934	0.830	0.686	0.897	0.915	0.904	0.796	0.932
SE-UNet	0.827	0.927	0.794	0.640	0.920	0.937	0.917	0.821	0.941
UNeXt	0.809	0.912	0.723	0.635	0.866	0.903	0.865	0.702	0.904
ResNet	0.830	0.853	0.589	0.585	0.933	0.937	0.910	0.807	0.958
DeepLab v3	0.852	0.937	0.827	0.668	0.896	0.931	0.908	0.809	0.938
SegNet	0.842	0.917	0.658	0.673	0.868	0.929	0.904	0.793	0.930
DeepPyramid	0.881	0.944	0.843	0.716	0.930	0.914	**0.933**	0.834	0.944
CE-Net	0.884	0.937	0.836	0.688	0.940	0.937	0.912	0.828	0.950
DSR-Net	**0.885**	**0.944**	**0.849**	**0.731**	**0.941**	**0.940**	0.910	**0.837**	**0.950**

The bold indicates the highest segmentation accuracy of this structure, and underline indicates the accuracy achieved by this model when the segmentation accuracy is not the highest.

**TABLE 4:** Segmentation results of each model (dice).

Model	Primary position				Gaze positions		
	Scleral	Iris	Pupil	LCP	Scleral	Iris	Pupil
U-Net	0.965	0.920	0.903	0.806	0.953	0.949	0.876
SE-UNet	0.904	0.962	0.881	0.940	0.967	**0.956**	0.892
UNeXt	0.892	0.953	0.831	0.766	0.948	0.926	0.814
ResNet	0.904	0.919	0.731	0.724	0.967	0.952	0.886
DeepLab v3	0.920	0.967	0.901	0.788	0.963	0.952	0.887
SegNet	0.913	0.956	0.778	0.798	0.962	0.949	0.876
DeepPyramid	0.936	0.961	0.910	0.827	0.965	0.955	0.903
CE-Net	0.937	0.967	0.909	0.809	**0.967**	0.953	0.899
DSR-Net	**0.938**	**0.969**	**0.914**	**0.839**	0.963	0.952	**0.923**

The bold indicates the highest segmentation accuracy of this structure, and underline indicates the accuracy achieved by this model when the segmentation accuracy is not the highest.

The comparative segmentation results of Model II and Model III are shown in Table 5. Although each classical model can achieve the segmentation of ocular structures, their handling of structural edges is less than ideal, with objects four and five being particularly noticeable. Building upon the U-shaped structure, the DSR-Net model addresses the distinct color characteristics of ocular color images by incorporating channel attention modules into the skip connections. This enhances segmentation accuracy while maintaining an acceptable inference speed, thereby improving spatial feature extraction capabilities. The improved segmentation accuracy subsequently elevates the precision of quantitative analysis.

**TABLE 5:** Visualization of partial segmentation results.

Object	Input	Label	DSR-Net	U-Net	UneXt	DeepLab v3	SegNet	Deep Pyramid	CE-Net
1	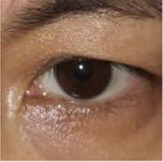	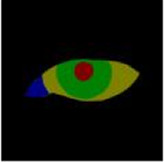	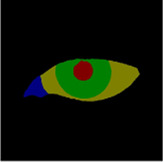	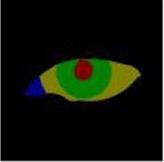	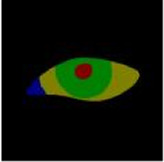	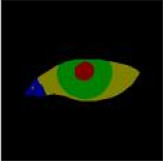	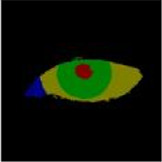	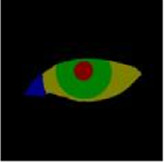	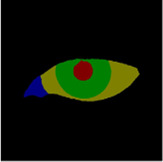
2	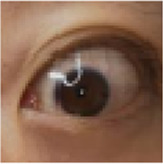	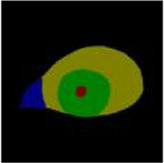	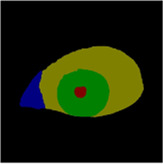	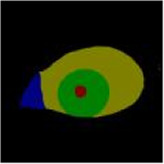	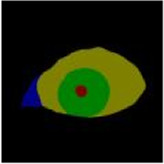	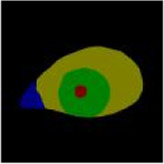	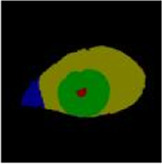	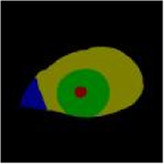	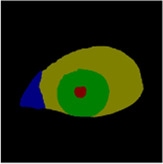
3	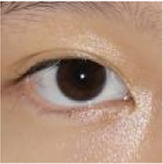	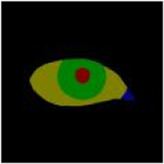	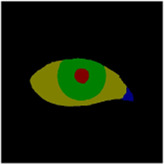	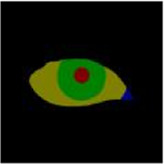	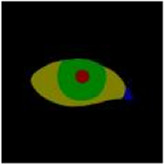	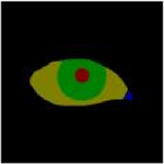	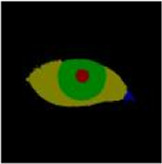	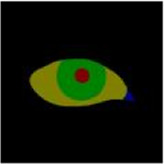	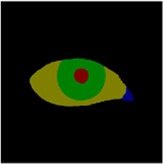
4	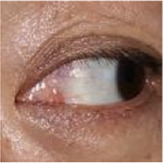	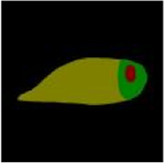	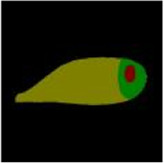	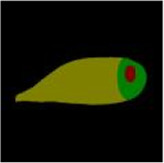	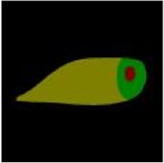	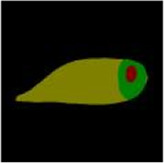	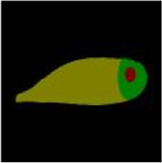	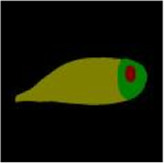	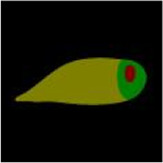
5	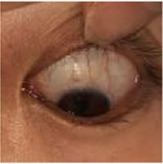	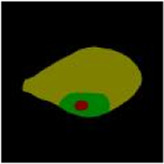	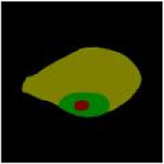	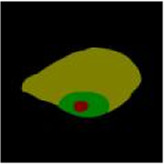	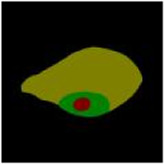	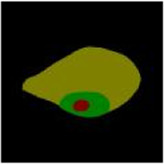	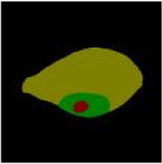	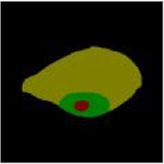	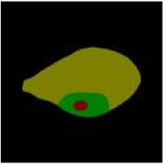

The DSR-Net achieved IoU scores of 81.1%, 80.1%, and 88.1% for the left eye, right eye, and drawing scale bar, respectively, in Module I, demonstrating its capability to effectively perform localization and cropping tasks. In Module II, the IoU scores for the sclera, iris, pupil, and plica regions in the primary position were 88.5%, 94.4%, 84.9%, and 73.1%, respectively. In Module III, the IoU scores for the sclera, iris, and pupil of the fixation position were 94.0%, 91.0%, and 83.7%, respectively. Although the IoU for the iris structure did not surpass Deep Pyramid’s 93.3%, DSR-Net demonstrated faster inference speed and superior performance in other aspects compared to Deep Pyramid. This indicates that the proposed model exhibits better overall performance, validating the effectiveness of the feature extraction capabilities of the DSR-Net model. Additionally, the Mean Pixel Accuracy (MPA) reached 94.1% and 95.0%, further confirming the high segmentation precision of the model.

**FIGURE 6:**
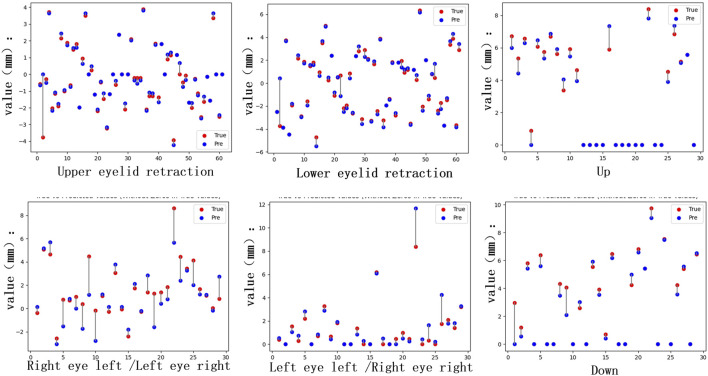
Quantitative differences. The distance quantification and actual distance discrepancies for Eyelid retraction and movement impairments in the upward, downward, leftward, and rightward gaze directions were analyzed. For the inward gaze position, the quantification involves the lacrimal punctum location in the primary position and the inner edge of the pupil in the current gaze position, requiring comprehensive consideration of both locations. This results in a higher number of individuals with significant discrepancies.

In the tasks of Model IV and Model V, the quantitative analysis achieved RMSE values of 0.847 for eye movement disorder, 0.667 for upper eyelid retraction, and 0.517 for lower eyelid retraction, As shown in Table 6, demonstrating good quantitative accuracy. The quantitative differences are illustrated in Figure 6.

**TABLE 6:** Quantitative result analysis.

Evaluation index	Eye movement disorder	Down	Up	Right eye left Left eye right	Left eye left Right eye right	Upper eyelid retraction	Lower eyelid retraction
RMSE	0.847	0.351	0.735	1.276	0.789	0.667	0.517
MSE	0.462	0.163	0.421	0.887	0.394	0.323	0.197
*R* ^2^	0.889	0.982	0.934	0.682	0.824	0.936	0.914

As shown in Table 7, the accuracy in the diagnostic tasks for eye movement disorder and upper and lower eyelid retraction reached 85.4%, 95.1%, and 87.0%, respectively, indicating that the diagnostic results are reliable. Due to individual differences in the scleral region, shadows between the eyelids and sclera are present in some patients, and the boundary between the iris and sclera is relatively distinct, resulting in slightly higher segmentation accuracy for the iris compared to the sclera. Because TED patients may have eye lesions such as swelling, segmenting the LCP region in the primary position is challenging. The diagnostic experiments for eyelid retraction and eye movement disorder in this paper are based on the segmentation results, which have good accuracy and can quickly and conveniently determine whether a patient has eyelid retraction and eye movement disorder, providing important assistance for the early diagnosis and prognostic treatment of TED.

**TABLE 7:** Evaluation indicators of diagnostic results.

Model	Eye movement disorder			Upper eyelid retraction			Lower eyelid retraction		
	ACC	*P*	TPR	ACC	*P*	TPR	ACC	*P*	TPR
U-Net	0.828	0.759	0.833	0.855	0.750	0.857	**0.887**	0.838	0.929
SE-UNet	0.762	0.708	0.715	0.838	0.720	0.857	0.854	0.787	0.928
UNeXt	0.825	0.759	0.833	0.919	0.904	0.857	0.725	0.627	0.999
ResNet	0.770	0.752	0.656	0.870	0.724	0.964	0.838	0.736	0.999
DeepLab v3	0.830	0.778	0.823	0.919	0.863	0.904	0.806	0.710	0.999
SegNet	0.834	0.756	0.882	0.951	**0.964**	0.857	0.709	**0.908**	0.999
DeepPyramid	0.834	0.774	0.843	0.919	0.863	0.904	0.867	0.800	0.999
CE-Net	0.826	0.765	0.833	0.951	0.875	**0.964**	0.854	0.756	0.999
DSR-Net	**0.854**	**0.843**	**0.843**	**0.951**	0.950	0.904	0.870	0.777	**0.999**

The bold indicates the highest segmentation accuracy of this structure, and underline indicates the accuracy achieved by this model when the segmentation accuracy is not the highest.

**FIGURE 7:**
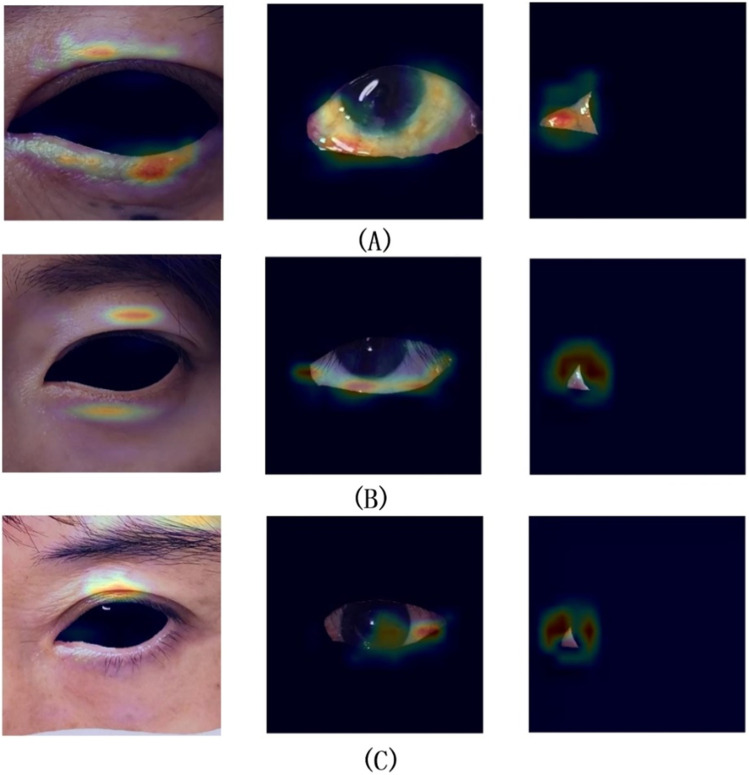
Groups **(A,B)** represent successful classification cases, while group **(C)** represents a failed classification case.

**FIGURE 8:**
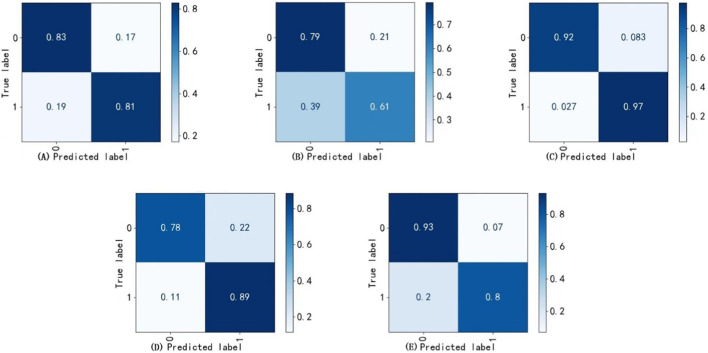
Confusion matrix. **(A)** Redness of eyelids, **(B)** Swelling of conjunctiva, **(C)** Redness of conjunctiva, **(D)** Swelling of eyelids **(E)** Swelling of caruncle or plica.

The best weights from Model II were selected to execute the task of Model VI, achieving the recognition and cropping of ocular structures for use as image input in the multi-label inflammation classification task of Module VII. TBRM-Net utilizes residual connections in the main branch to learn deep features and integrates CNN with Transformer through the MobileViT module for feature extraction. It employs fewer parameters in the main branch to integrate local and global features of the input tensor, and fuses convolutional features between each module of the main branch and the auxiliary branch, enabling sufficient dimensional feature interaction. This feature extraction method demonstrates superior capability compared to other classical networks. As shown in Figure 7 by Grad-CAM, it can better focus on the structural regions where inflammation is present. In the tasks of Model VI and Model VII, the classification evaluation results of TBRM-Net for the five types of inflammation involved in CAS are presented in Table 8. The accuracy (Acc) for Redness of Eyelids, Swelling of Eyelids, Redness of Conjunctiva, Swelling of Conjunctiva, and Swelling of Caruncle or Plica reaches 81.8%, 78.8%, 90.6%, 73.5%, and 83.9%, respectively, with an average accuracy of 81.7%. The confusion matrix is shown in Figure 8. The segmentation of the structures affected by inflammation in the eye can effectively exclude interfering features, demonstrating superior classification performance in multi-label classification tasks.

**TABLE 8:** Acc of two label classification of structured data sets in dataset1.

Acc	Eyelid Inflammation		Conjunctival Inflammation		LCP	Mean
	Redness of eyelids	Swelling of eyelids	Redness of conjunctiva	Swelling of coniunctiva	Swelling of caruncle or plica	
Resnet34	0.721	0.772	0.826	0.707	0.843	0.773
Resnet50	0.728	**0.829**	0.834	0.697	**0.846**	0.786
Convnet	0.606	0.723	0.840	0.620	0.770	0.711
Shufflenet_v2	0.642	0.770	0.901	0.685	0.792	0.758
Mobilenetv2	0.723	0.694	0.827	0.621	0.828	0.739
MobileVit	0.727	0.705	0.820	0.650	0.755	0.731
TBRM-Net	**0.818**	0.788	**0.906**	**0.735**	0.839	**0.817**

The bold indicates the highest segmentation accuracy of this structure, and underline indicates the accuracy achieved by this model when the segmentation accuracy is not the highest.

## 4 Discussion

TED presents with complex clinical manifestations affecting the extraocular muscles, eyelids, and other tissues (Wiersinga and Bartalena, 2002), which can lead to eyelid retraction and eye movement disorder. Consequently, accurate diagnosis typically requires the expertise of experienced orbital physicians. However, there is currently a relative scarcity of specialized orbital physicians, which poses challenges for the diagnosis, evaluation, and management of TED. Internationally and domestically recognized diagnostic standards and guidelines already exist (Bartalena et al., 2021), and characteristic signs can be identified through imaging studies. This provides a foundation for the application of artificial intelligence (AI) in auxiliary diagnosis. Currently, the majority of studies employ professional digital cameras to capture eye images from patients and develop artificial intelligence-based diagnostic models. The use of smartphones for eye image acquisition is typically combined with portable slit lamps or portable fundus camera devices (Vilela et al., 2024). However, there has been no reported research on utilizing smartphone-collected eye images specifically for the development of TED diagnostic models. In this study, five-dimensional eye images of TED patients were successfully captured using smartphones, enabling not only the documentation of characteristic ocular signs but also the detection of eye movements. Two semantic segmentation data sets were successfully constructed: primary position and fixation position, and inflammation classification data set, comparing the segmentation performance of different deep learning models on the ocular structures of TED patients and their classification efficacy regarding ocular inflammation.

The DSR-Net model c in this study captures multi-level and multi-scale semantic features, enhancing the ability to extract channel features from color images based on the learning of spatial information, The diagnosis of related symptoms is based on eye image segmentation. TBRM-Net employs a dual-branch strategy to extract and fuse inflammation features at different scales. Furthermore, this study innovatively introduced a quantitative recognition model for eyelid retraction and eye movement disorder, which can identify and quantitatively evaluate these key diagnostic indicators based on the semantic segmentation results of the eye structure. The model achieves diagnostic accuracy rates of 85.4%, 95.1%, and 87.0% for TED eye movement disorders and upper and lower eyelid retraction, respectively. Segmentation preprocessing was performed on the structures involved in inflammation. In the classification of Redness of Eyelids, Swelling of Eyelids, Redness of Conjunctiva, Swelling of Coniunctiva, and Swelling of Caruncle or Plica, the Accuracy reaches 81.8%, 78.8%, 90.6%, 73.5%, and 83.9%, respectively, allowing for rapid and convenient clinical assistance in diagnosing TED conditions. According to the EUGOGO guidelines, the degree of eyelid retraction and the severity of diplopia are key factors in evaluating the severity of TED (Bartalena et al., 2016; Bartalena et al., 2021; Bartalena et al., 2008). The quantitative recognition model for eyelid retraction, eye movement disorders, and inflammation classification established in this study provides valuable assistance in assessing the severity of TED in patients.

### 4.1 Standardized acquisition of facial images

In the clinical management of TED patients, the changes of ocular inflammatory signs, eyelid retraction, and ocular motility disorders are very important to evaluate. The assessment results given by doctors with different experience may be different. AI-assisted diagnostic system can achieve standardized assessment, improve diagnostic efficiency and reduce human error. The standardization of AI diagnostic system is based on the standardization of image data acquisition. In this study, a standardized mode of facial image acquisition was established based on the detailed criteria of EUGOGO guidelines for the assessment of TED. The standard sets the image size, shooting light, Angle, face to image ratio, image background and other factors in detail, and tries to ensure that the photo effect is similar to the actual scene when the doctor’s clinical diagnosis is made. Compared to Xiao et al. (2022), who used professional digital SLR cameras (SONY ILCE-7M2) to capture eye images, this study employed a smartphone for image collection to enhance the universality and accessibility of the model. The combination of smartphone and AI is also a research hotspot, especially in the screening of eye diseases with high blinding rate such as diabetic retinopathy, retinopathy of prematurity, glaucoma and age-related macular degeneration (Sharafeldin et al., 2018), which has shown potential and advantages. In this study, the five-dimensional external observations of patients were standardized collected by smartphone and used as the data set for model training. The screening model established by this method may have a wider applicability and more audience groups.

The acquisition of stable head position images is helpful to establish a standardized recognition model and achieve accurate judgment of eyelid retraction and ocular motility disorders in TED patients. In two studies that established eye movement detection models based on the facial images of TED patients and normal people, the patients were orally asked to maintain a stable head position during the photo taking (Smith et al., 2023; Lou et al., 2022). We considered that due to the patient’s compensatory head position and cooperation degree, it may be difficult to maintain a stable head position when the eyes move to different eye positions in the actual photo taking. The position of the patient’s head is very important in the process of image acquisition. The rise and fall of the jaw and the tilt of the head will affect the judgment of the results. A study by Scheetz et al. (2021) showed that compensatory head position and head tilt would lead to compensatory eye movement rotation in patients, and the amplitude of rotation was positively correlated with the degree of head tilt. Therefore, the use of a special head-fixing device in this study can ensure the stability of the patient’s head position and avoid the distortion of the measurement results caused by the change of head position, so as to improve the stability and consistency of the image. The device, which has been patented at the same time, can also be used for photographic measurement of normal human eye movements.

The key to establish the quantitative identification detection model is to set the scale identification when the image is collected. There are differences in the quantitative scales used in different AI quantitative models. Van Brummen et al. (2021) developed an automatic measurement model of key eyelid parameters based on deep learning, including the distance between the upper eyelid margin and the pupil center, the distance between the lower eyelid margin and the pupil center, etc. In the process of data collection, a scale bar with a scale of 10 mm was placed on the forehead of the patient, which was used as a scale bar for pixel conversion in the data processing stage. Lou et al. (2022) studied an analysis method for automatic measurement of eye movements based on facial photographs and deep learning techniques, which could objectively assess the amplitude of eye movements. In this study, a circular marker patch with a diameter of 10 mm was pasted on the forehead of volunteers during facial image acquisition as a reference for quantification. Referring to the quantitative method of the above study, a ruler was set on the side of the special fixation device of the head during the external observation of the patient’s face. In order to improve the contrast, it was set as a small black and white cell, each cell was 5 mm. The quantitative signs in this study were fixed in position, and there was no need to paste or place other reference signs on the forehead of the patient. It simplifies the photography process while obtaining reliable and stable data.

### 4.2 Model identification performance

#### 4.2.1 The recognition performance of quantitative model V for eyelid retraction

The segmentation model established in this study demonstrated strong capability in recognizing the sclera, iris, and pupil, with IoU scores of 0.885, 0.944, and 0.849, respectively, indicating a relatively high level of AI image segmentation performance and efficiency. This provided a solid foundation for the subsequent development of a quantitative model for eyelid retraction. The designed Model V achieved ACC scores of 0.951 and 0.870 for upper and lower eyelid retraction, respectively, and RMSE values of 0.667 and 0.517 in quantitative evaluation, reflecting a high level of quantification accuracy and interpretability.

The TED intelligent diagnostic system based on facial images developed by Xiao et al. (2022) can accurately identify eyelid retraction, with a sensitivity of 0.87 and a specificity of 0.88. The network used for the development of this model is ResNet50, and the dataset consists of 1,560 patients’ eye appearance photos, with the model being trained for 100 epochs. Our model achieved accuracies of 0.951 and 0.870 for upper and lower eyelid retraction, respectively, and Precisions of 0.950 and 0.777, respectively, using a dataset of 153 patients’ eye appearance photos. During the model training process, we continuously adjusted parameters and conducted training for 200 epochs. The diagnostic performance of our model for eyelid retraction is comparable to the results of Xiao et al. (2022), and is slightly stronger in the diagnostic capability for patients with eyelid retraction. This may be related to the number of times we trained the model, the use of the DSR-Net network which is more optimized for edge recognition accuracy, and the fact that the diagnosis of eyelid retraction is based on the quantitative assessment of eye structure morphology, which is more explanatory than classification evaluation based on a large amount of data.

Accurate measurement of eyelid retraction is crucial for the diagnosis of TED, grading of disease severity, surgical design for upper eyelid retraction, and evaluation of treatment efficacy. The eyelid retraction quantification model established in this study can not only perform qualitative diagnosis but also quantitative assessment, with an accuracy of recognition consistent with the method proposed by Shao Ji et al. (2023). The model established in this study can quantify eyelid retraction of any degree and has certain advantages in measuring mild TED patients. In evaluating the changes in the condition of TED patients and follow-up of postoperative efficacy, where observation of eyelid retraction is required, this model can perform continuous quantitative monitoring.

#### 4.2.2 The recognition performance of quantitative model IV for eye movement disorder

Extraocular muscles are the main sites involved in the development of TED, and ocular movement disorders are caused by inflammatory cell infiltration. In the later stage, rapid fibrosis leads to reduced muscle elasticity and then restrictive strabismus (Eckstein et al., 2018). TED is the most common cause of enlargement and dysfunction of extraocular muscles, mainly involving the inferior and medial rectus muscles, which can lead to horizontal and vertical strabismus, leading to diplopia and seriously affecting the quality of life of patients (Savino et al., 2020). Ocular motility disorders play a very important role in the diagnosis and severity assessment of TED (Bartalena et al., 2016; Bartalena et al., 2021). Therefore, clinical evaluation of ocular motility disorders can help to judge the severity of TED, and then conduct personalized treatment for patients.

The clinical examination of eye movements in TED patients largely depends on the experience of doctors, and there is no standardized tool for detecting eye movements at present (Hanif et al., 2009). The most commonly used method in clinical practice is that the examiner uses a flashlight to guide the patient to fixate on eight eye positions. According to the range of eye movement, and the position relationship with the anatomical landmarks such as the inner and outer canthus, the upper and lower lacrimal points, the disorder of eye movement is determined (Vivian and Morris, 1993). The method is simple and easy to operate and has strong clinical applicability, but it is easily affected by the experience of the examiner and the change of the patient’s head position. With the increasingly powerful function of deep learning technology for automatic image segmentation, it can segment and identify structures such as optic disc and blood vessels according to anatomical landmarks. At present, the commonly used eye movement examination methods are also based on fixed anatomical landmarks. Therefore, it is possible to apply deep learning to the evaluation of eye movement disorders in TED. In this study, the five-dimensional external observation of patients was used to establish a recognition model of eye movement disorders based on deep learning network. This model can improve the efficiency of eye movement examination and evaluate the changes of TED patients. The external facial views of TED patients and normal healthy people used in this study were taken by smart phones with the assistance of head fixation devices, which not only considered the influence of the patient’s head position on the eye position, but also considered the universality and portability of the model in the future. At present, there is no report on the evaluation of the eye movements of TED patients based on external facial observation and deep learning technology. This study is innovative and practical.

Lou et al. (2022) developed an automatic model for measuring ocular motility based on nine-directional facial photographs using a convolutional neural network. This study included 207 healthy individuals as a test set and compared the results with manual measurements, showing good consistency between the two methods. However, the model has not been applied to the recognition of patients with ocular motility disorders, and its detection performance requires further clinical validation. The ocular motility disorder model established in this study performed well in a test set composed of healthy individuals and TED patients, with an Accuracy of 0.854 and a Precision of 0.843, indicating excellent diagnostic performance. The mean RMSE for quantitative diagnosis was 0.847, suggesting a high level of interpretability based on quantitative diagnosis. This study included both healthy individuals and TED patients with ocular motility disorders, ensuring good data balance.

#### 4.2.3 The recognition effect of ocular inflammatory signs model

Currently, there are only a few reports on AI recognition of TED signs based on facial images. No studies have been reported on AI recognition using facial photographs of TED patients taken with smartphones. The course of TED typically begins with an inflammatory active phase, during which patients generally exhibit ocular inflammatory signs such as conjunctival congestion and edema, eyelid congestion and edema, and swelling of the lacrimal caruncle folds. In severe cases, patients may go blind due to exposure keratopathy and compressive optic neuropathy (Yamada et al., 2000; Rundle and Wilson, 1945). The inflammatory active phase usually lasts for 18 months to 2 years before stabilizing and entering the fibrotic stage (Bartalena et al., 2020). Early diagnosis and aggressive treatment can alter the course of the disease and reduce the incidence of severe cases (Kauppinen-Mäkelin et al., 2002). Therefore, early diagnosis and treatment of TED have become a focus of research.

The TED intelligent diagnostic system developed by Xiao et al. (2022) is capable of detecting various signs of the disease. The model’s AUC for detecting Swelling of Eyelids, Redness of Eyelids, Redness of Conjunctiva, and Swelling of Conjunctiva are 0.90, 0.94, 0.91, and 0.60, respectively. The model established in this study identifies and classifies the structures affected by inflammation, with Accuracy values of 0.818, 0.788, 0.906, 0.735, and 0.839 for Redness of Eyelids, Swelling of Eyelids, Redness of Conjunctiva, Swelling of Conjunctiva, and Swelling of Caruncle or Plica, respectively, which enhances the model’s performance by excluding interference from inflammation of unrelated structures during feature extraction.

We have proposed an auxiliary diagnostic system for TED that can quantitatively diagnose disorders of ocular motility and eyelid retraction, classify and recognize inflammation of CAS in relevant structures, and compare the results with a test dataset. This system enables rapid and non-invasive diagnosis based on ocular images, showcasing potential applications in the clinical diagnosis of TED.

This study has certain limitations. The model demonstrates good diagnostic performance for inflammation, eyelid retraction, and eye movement disorders, but exhibits relatively lower precision for lower eyelid retraction, and poorer quantitative diagnostic results for right eye leftward rotation and left eye rightward rotation. The diagnostic accuracy for conjunctival edema is also slightly low. To enhance the model’s generalizability, we will expand the dataset and conduct external validation using relevant multi-center datasets. Future efforts will focus on further tuning the model to enhance its ability to extract features and broaden its application scope. Additionally, integrating eye images with other data, such as slit-lamp anterior segment photographs and patient complaints, to assist in the diagnosis of TED will be a key area of emphasis in our future research.

## Data Availability

The raw data supporting the conclusions of this article will be made available by the authors, without undue reservation.
